# A Computational Model of Kidney Function in a Patient with Diabetes

**DOI:** 10.3390/ijms22115819

**Published:** 2021-05-29

**Authors:** Rui Hu, Anita Layton

**Affiliations:** 1 Department of Applied Mathematics, University of Waterloo, Waterloo, ON N2L 3G1, Canada; rui.hu@uwaterloo.ca; 2 Department of Biology, Cheriton School of Computer Science, and School of Pharmacology, University of Waterloo, Waterloo, ON N2L 3G1, Canada

**Keywords:** SGLT2 inhibitors, epithelial transport, sodium transport, glucose transport, natriuresis, diuresis

## Abstract

At the onset of diabetes, the kidney grows large and the glomerular filtration rate becomes abnormally high. These structural and hemodynamics changes affect kidney function and may contribute to the development of chronic kidney disease. The goal of this study is to analyze how kidney function is altered in patients with diabetes and the renal effects of an anti-hyperglyceamic therapy that inhibits the sodium-glucose cotransporter 2 (SGLT2) in the proximal convoluted tubules. To accomplish that goal, we have developed a computational model of kidney function in a patient with diabetes and conducted simulations to study the effects of diabetes and SGLT2 inhibition on solute and water transport along the nephrons. Simulation results indicate that diabetes-induced hyperfiltration and tubular hypertrophy enhances Na^+^ transport, especially along the proximal tubules and thick ascending limbs. These simulations suggest that SGLT2 inhibition may attenuate glomerular hyperfiltration by limiting Na^+^-glucose transport, raising luminal [Cl^−^] at the macula densa, restoring the tubuloglomerular feedback signal, thereby reducing single-nephron glomerular filtration rate.

## 1. Introduction

The prevalence of diabetes is rising worldwide, currently estimated to be 9.3% (463 million people) and expected to reach 10.2% (578 million) by 2030 and 10.9% (700 million) by 2045 [1]. In developed countries, type 2 diabetes is among the most common causes of chronic kidney disease [2] and a major contributor to cardiovascular disease [3]. Even though the pathways that link diabetes to chronic kidney disease remain incompletely understood, diabetes is known to induce pathophysiological changes in the kidneys. At the very onset of diabetes, the kidney grows large and the glomerular filtration rate (GFR) becomes supranormal [4]. These structural and hemodynamic changes affect kidney function and may eventually lead to chronic kidney disease.

Throughout the animal kingdom kidneys are known primarily for their function as filters, removing metabolic wastes and toxins from the blood for excretion in the urine. But in mammals, kidneys specialize to serve various other essential regulatory functions, including water, electrolyte and acid-base balance [5]. In humans, the pair of kidneys are located in the abdominal cavity, with one on each side of the spine. A mammalian kidney can be divided into an outer region (cortex) and an inner region (medulla). Each of the human kidneys contains about a million glomeruli, which are clusters of capillaries that each receive blood from individual afferent arterioles branching off intra-renal arteries. Driven by vascular hydrostatic pressure, a fraction of the water and solutes in that blood is filtered through the glomerulus and becomes the tubular fluid of the nephron. The nephrons adjust the content of the glomerular filtrate, via absorptive and secretive processes, mediated by membrane transporters and channels on the renal tubular epithelial cells. Thus, what begins at the glomerulus as an ultrafiltrate of plasma is transformed into urine at the end of the nephrons [5]. Regulation of the epithelial transport processes that match urine output to both intake of fluids and solutes as well as to waste product production is the subject of a large body of experimental and theoretical effort [6,7,8,9]. Computational models have been developed to unravel the renal solute and water transport processes in humans [10] and rats, under dietary [11,12,13] and therapeutic [14,15,16] manipulations, and under pathophysiological conditions [17,18,19]. 

As previously noted, diabetes is a major risk factor for kidney disease. That is one among many reasons for patients with diabetes to attain glyceamic control. A new class of anti-hyperglycaemic drugs are the sodium-glucose cotransporter 2 (SGLT2) inhibitors, which enhance urinary glucose excretion and attenuate postprandial increases in blood glucose by targeting its reabsorption along the early proximal tubule [20]. As a cotransporter, SGLT2 mediates glucose transport in a process that is coupled with Na^+^ transport; thus, the inhibition of SGLT2 also reduces proximal tubular Na^+^ and fluid reabsorption, and induces natriuresis and diuresis. Indeed, in addition to its anti-hyperglycaemic effect, SGLT2 inhibitors have been reported to reduce blood pressure and protect diabetic patients from heart failure [21,22]. The renal, metabolic, and cardiovascular impacts of SGLT2 inhibition have been reviewed in Refs. [23,24]. 

We have previously conducted model simulations to investigate kidney function in diabetes and the renal effects of SGLT2 inhibition [15,16,17]. However, the computational model used in those studies were based on a rat kidney. As such, even though some of the model predictions are consistent with observations in patients with diabetes, the translational value of those findings may still be limited. Thus, the goal of the present study is to analyze kidney function in (human) patients with diabetes and the effects of SGLT2 inhibition, using a computational model of a human kidney that we recently published [10,25].

## 2. Results

### 2.1. Kidney Function under Non-Diabetic and Diabetic Conditions

**Figure 1:**
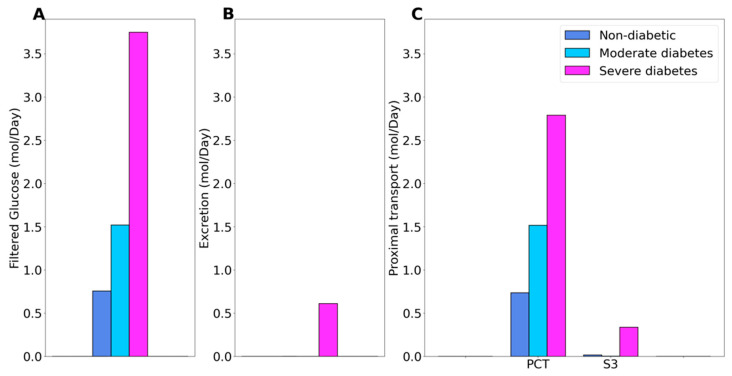
Glucose handling under non-diabetic, moderate diabetes, and severe diabetes conditions. (**A**), filtered glucose. (**B**), predicted glucose excretion. (**C**), glucose transport along the proximal convoluted tubule (PCT) and S3 segment.

**Figure 2:**
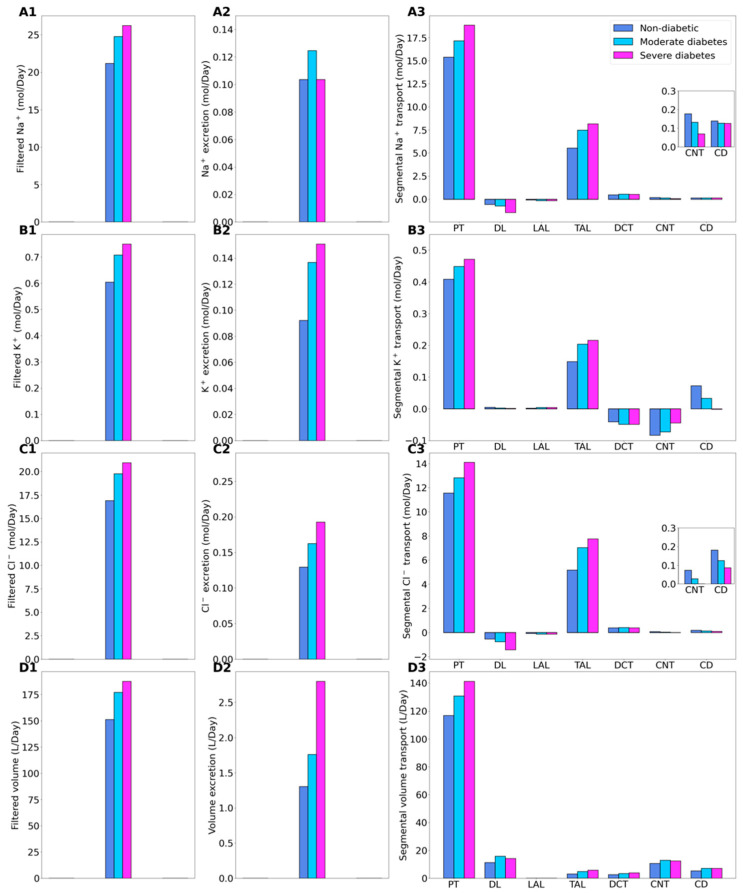
Renal transport of key solutes and water under non-diabetic, moderate diabetes, and severe diabetes conditions. Left column, filtered Na^+^ (**A1**), K^+^ (**B1**), Cl^−^ (**C1**), and water (**D1**). (**A2**–**D2**) excretion rates. (**A3**–**D3**) corresponding segmental transport.

We compare solute and water transport along the nephrons in a non-diabetic and diabetic kidney. Key results are summarized in Figure 1 and Figure 2. In these simulations, we mimic the renal effects of diabetes as described in Materials and Methods. In particular, diabetes induces glomerular hyperfiltration and tubular hypertrophy. Elevated GFR is reflected on filtered solute loads, whereas tubular hypertrophy is reflected in the enhanced transport; see below. 

Under non-diabetic conditions, essentially all filtered glucose is reabsorbed along the proximal tubules, with the proximal convoluted tubule and S3 segment reabsorbing 97% and 2.6% of filtered glucose, respectively. See Figure 1C. In moderate diabetes simulations, plasma glucose concentration is assumed to increase from 5 to 8.6 mM, which together with the elevated GFR yields a filtered load of 1.52 mol·day^−1^ glucose. Despite filtered glucose being approximately doubled, the proximal tubule glucose transport has yet to be overwhelmed, with the proximal convoluted tubule and S3 segment reabsorbing 99.6% and 0.4% of the filtered glucose, respectively, leaving essential no glucose in the urine, consistent with the observed absence in glucosuria in patients with moderate diabetes [26]. In severe diabetes simulations, plasma glucose is further elevated to 20 mM, resulting in glucose filtered load of 3.75 mol·day^−1^ glucose, which exceeds the glucose transport capability of the proximal tubule. The fractional reabsorption of glucose by the proximal convoluted tubule and S3 segment is predicted to be 73.7% and 9.1%, respectively. Downstream segments do not possess significant glucose transport capacities. As such, the diabetic model predicts absolute and fractional glucose excretion as 0.6 mol·day^−1^ and 16%, respectively. 

Relative to the non-diabetic kidney, the moderate diabetic model assumes a 10% increase in GFR and thus filtered Na^+^, which yields a corresponding 10% increase in total Na^+^ transport. The increase in Na^+^ reabsorption is largest in tubular segments where diabetes induces significant increases in the expression of Na^+^ transporters. In particular, Na^+^ transport increases by 11.8% along the proximal convoluted tubules, where hyperfiltration-induced changes in the torque augment the density of all transcellular transporters. Diabetes also enhances the density of NKCC2 in the medullary thick ascending limbs [17], resulting in an 35% increase in Na^+^ reabsorption. The enhanced Na^+^ transport essentially compensates for the elevated filtered Na^+^ load in diabetes to yield Na^+^ excretion similar to a non-diabetic kidney (Figure 2, panels A1–A3). Elevated Na^+^ reabsorption is followed by increases in the reabsorption of Cl^-^ and water (Figure 2, panels C3 and D3). Compared to the non-diabetic case, urinary Cl^-^ excretion is predicted to be 25% higher in diabetes, whereas urine output is 35% higher (Figure 2, panels C2 and D2). Similar to Na^+^, the higher filtered K^+^ load enhances its tubular reabsorption along the proximal tubules and thick ascending limbs (Figure 2(B3)). These competing factors result in kaliuresis, with K^+^ excretion predicted to be 48% higher in diabetes (Figure 2(B2)).

In the severe diabetes model, GFR and filtered Na^+^ both increase by 24%, resulting in enhanced Na^+^ transport, notably by 22.5% along the proximal tubules and 47.4% along the thick ascending limbs. As in the moderate diabetes case, the enhanced Na^+^ transport essentially compensates for the elevated filtered Na^+^ load in diabetes to yield Na^+^ excretion similar to a non-diabetic kidney (Figure 2, panels A1–A3). Model predictions of Cl^-^ transport are analogous as those for the moderate diabetes case (Figure 2(C2)). Compared to the non-diabetic case, urinary Cl^-^ excretion is predicted to be 48.8% higher in diabetes. Model predicts more severe diuresis, with urine output predicted to be 115% higher than the non-diabetic case (Figure 2(D2)), and more severe kaliuresis, with K^+^ excretion predicted to be 63.4% higher (Figure 2(B2)).

### 2.2. SGLT2 Inhibition in a Non-Diabetic Kidney

**Figure 3:**
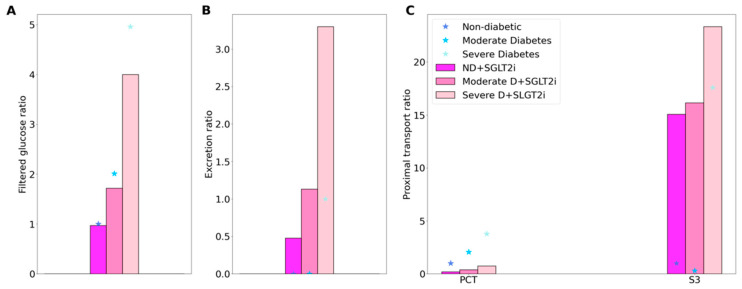
Glucose handling under non-diabetic (ND) and diabetic conditions (D), with SGLT2 inhibition (SGLT2i, denoted by color bars) and without SGLT2 inhibition (denoted by asterisks). (**A**), filtered glucose, normalized by ND (no SGLT2i) value. (**B**), glucose excretion, normalized by D (no SGLT2i) value. (**C**), glucose transport along the proximal convoluted tubule (PCT) and S3 segment.

In the non-diabetic setting, SGLT2 blockade has been found to induce a minor GFR reduction: by 3% in non-diabetic subjects receiving canagliflozin or dapagliflozin for 4 days [17]. Thus, we assume that in the acute inhibition simulation a 3% reduction in the SNGFR of all nephrons. Also, in the non-diabetic setting, SGLT2 blockade has been reported to yield a urinary excretion of glucose that is ~45% of its filtered amount [27]. In the present study, we assume 90% inhibition of SGLT2 in all nephrons, which results in the excretion of 40% of the filtered glucose [27]. The model predicts that a small fraction (14.5%) of the filtered glucose load is reabsorbed along the proximal convoluted tubule segments, mediated by the remaining SGLT2 and also via the paracellular route, and 39.7% in the S3 segment, most of which across SGLT1. See Figure 3. 

**Figure 4:**
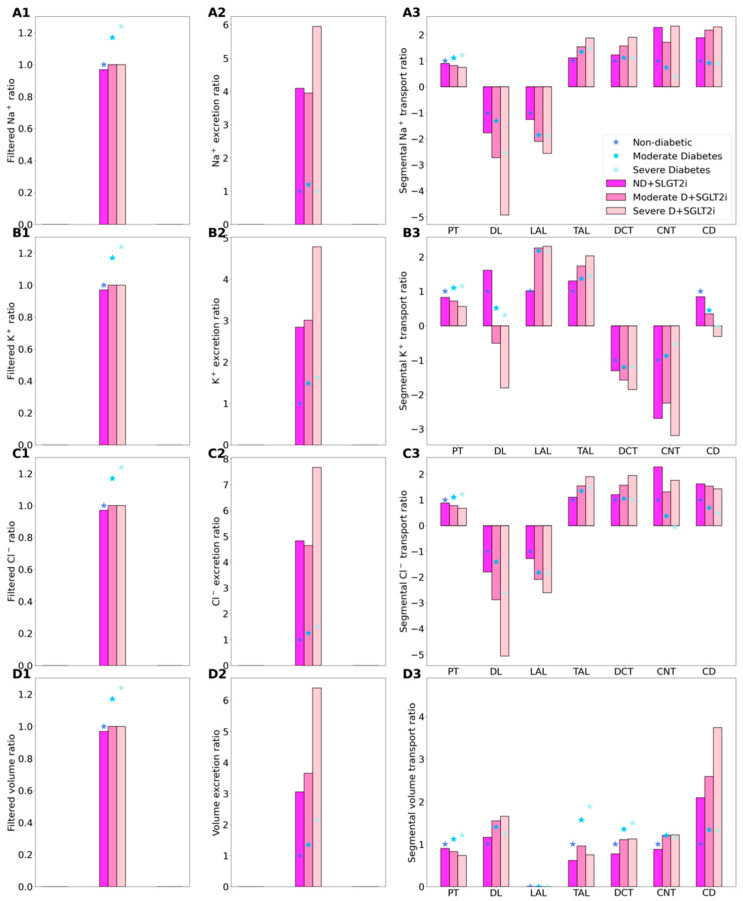
Renal transport of key solutes and water under non-diabetic (ND) and diabetic (D) conditions with SGLT2 inhibition (SGLT2i, denoted by color bars) and without SGLT2 inhibition (denoted by asterisks). Left column, filtered Na^+^ (**A1**), K^+^ (**B1**), Cl^−^ (**C1**), and water (**D1**), normalized by the corresponding ND (no SGLT2i) value. (**A2**–**D2**) normalized excretion rates. (**A3**–**D3**) corresponding segment transport, normalized by each segment’s ND absolute value.

In Figure 3 and Figure 4, the non-diabetic and diabetic cases without SGLT2 blockade (denoted “ND” and “D”) results are the same as those in Figure 1 and Figure 2, but presented differently. Instead of actual values, we normalize the filtration and segmental transport rates by their respective ND values. For excretion rates, we normalize by the D values, because under non-diabetic conditions glucose excretion is essentially zero. By computing these ratios, we can better understand how much each quantity changes in diabetes and following SGLT2 inhibition. However, cross comparison (e.g., between filtration and excretion) is meaningless due to their different reference values.

Consider again the non-diabetic SGLT2 inhibition results. As we previously described [16,17], SGLT2 inhibition elicits osmotic diuresis in the proximal tubule, thereby reducing passive transport via the paracellular route in that segment. The high luminal flow conversely stimulates active transport (via torque-induced increases in transcellular transporter expression [18]), but the reduction in passive transport is greater, so that net Na^+^ reabsorption decreases in the proximal tubule, by 10.3%. Simulation results suggest that the reduction in the proximal tubule Na^+^ transport is then partly compensated for downstream, particularly beyond the medullary thick ascending limbs (Figure 4(A3)). The elevated Na^+^ reabsorption along the connecting tubules is accompanied by enhanced K^+^ secretion (Figure 4(B3)). Consequently, SGLT2 inhibition in a diabetic kidney induces diuresis, natriuresis, and kaliuresis, with urine output increases by 207%, Na^+^ excretion by 308%, and K^+^ excretion by 184% (Figure 4, panels A2–D2).

### 2.3. SGLT2 Inhibition in a Diabetic Kidney

SGLT2 inhibition attenuates diabetic-reduced hyperfiltration [28]. Thus, when simulating the effects of SGLT2 blockade, we lower GFR to its non-diabetic level of 151.2 L·day^−1^. We consider an acute administration, so plasma glucose concentration is kept at 8.6 and 20 mM in the moderate and severe diabetes cases, respectively. First consider glucose transport in the moderate diabetes case. With the above GFR assumption, SGLT2 inhibition reduces the filtered load of glucose from 1.52 to 1.3 mol·day^−1^. Glucose excretion is predicted to be 0.69 mol·day^−1^, that is, 53.1% of filtered glucose. The predicted glucose excretion is within the range of reported values [29]. The proximal convoluted tubule and S3 segments reabsorb 0.23 and 0.38 mol glucose·day^−1^, respectively. In comparison, in the absence of SGLT2 inhibition, the proximal convoluted tubule and S3 respectively reabsorb 1.51 and 0.01 mol glucose·day^−1^. See Figure 3.

In the severe diabetes case, glucose excretion is predicted to be 2.02 mol·day^−1^ (Figure 3B), which corresponds to 66.8% of filtered glucose, within the range of reported values [29]. The proximal convoluted tubule and S3 segments reabsorb 0.5 and 0.5 mol glucose·day^−1^, respectively, compared to the respective rate of 2.8 and 0.34 mol glucose·day^−1^ without SGLT2 inhibition (Figure 3C) It is noteworthy that whereas there is glucose secretion across the tight junction in the absence of treatment, the paracellular pathway mediates glucose reabsorption when SGLT2 is inhibited (owing to higher luminal glucose concentration). Altogether these results suggest that, with 90% inhibition of SGLT2, SGLT1 barely compensates for the blockade of SGLT2 in diabetes, because hyperglycemia and the increased tubular glucose load already consume the full transport capacity of SGLT1 in the absence of SGLT2 inhibition.

SGLT2 inhibition is predicted to significantly lower Na^+^ transport, primarily because it normalizes GFR and filtered Na^+^ load. Our simulations suggest that, as in non-diabetic case, blocking SGLT2 causes osmotic diuresis in the proximal tubule, thereby reducing paracellular transport. In fact, the model predicts that the direction of paracellular Na^+^ transport is reversed in S3: owing to osmotic diuresis, the luminal-to-interstitial Na^+^ concentration gradient favors Na^+^ secretion into the lumen via the tight junctions. Consequently, in the moderate diabetes case, Na^+^ excretion increases by 2.2 folds and urine output by 1.7 folds; in the severe diabetes case, Na^+^ excretion and urine output increase by 4.9 and 1.9 folds, respectively (Figure 4, panels A2 and C2). The higher Na^+^ flow along the distal tubular segments raises K^+^ secretion, increasing K^+^ excretion by 1.02 and 1.9 folds, in the moderate and severe diabetes cases, respectively (Figure 4, panels B2). The predicted increases, especially in urine output, are higher than reported values [30]. Excessive urine output may result in volume depletion, which in turn activate mechanisms to reduce urine production. Those mechanisms are not represented in the model, which may explain the high urine output and excretion predictions.

## 3. Discussion

The main goal of this study is to extend a computational model of the human kidney, and to apply that model to study the effects of diabetes and SGLT2 inhibition on solute and water transport along the nephrons. Diabetes induces glomerular hyperfiltration and tubular hypertrophy [31]. As a consequence, diabetes increases the reabsorption of Na^+^ and glucose via the sodium-glucose cotransporter SGLT2 in the early proximal tubule of the renal cortex, and to a lesser extent, via SGLT1 in the S3 segment of the renal medulla. The elevated solute and water transport loads lead to an increase in Na^+^ transport (T_Na_). A previous modeling study of the rat kidney [17] predicted a 50% increase in overall T_Na_, with a disproportional increase in active T_Na_ (64%). Active T_Na_ is mediated by Na^+^/K^+^-ATPase and requires oxygen consumption. Diabetes increases fatty acid metabolism, resulting in the reduction of metabolic efficiency [32], including an increase in the amount of oxygen required to transport a given amount of Na^+^. As a result, oxygen consumption to support active T_Na_ was predicted to almost double (88% increase [17]). 

The present human kidney model predicts that the largest diabetes-induced increases in T_Na_ occur along the proximal tubules and thick ascending limbs (Figure 2(A3)). While the proximal convoluted tubules reside in the well perfused renal cortex, the S3 and medullary thick ascending limbs reside in the outer medullary, where the oxygen tension is substantially lower than in the cortex [33,34]. Indeed, these segments are known to be vulnerable to hypoxic injury. As such, the diabetes-induced increase in metabolic demand may be particularly stressful for these segments. Taken together, the present model predicts, in the moderate and severe diabetes cases, a 17% and 24%, respectively, increase in overall T_Na_ and a 29.4% and 37.5% increase in active T_Na_. The extent of hyper-glycemia in the severe diabetes case is similar to the rat model [17]. However, the present model predicts a smaller increase in T_Na_. That difference can be attributed to the smaller diabetes-induced GFR and thus filtered Na^+^ load increases in humans. Nonetheless, the higher active T_Na_, together with the diabetes-induced reduction in metabolic efficiency, likely yield a substantial increase in oxygen demand. Without a corresponding increase in oxygen supply, the higher metabolic demand may result in renal hypoxia, which is thought to be an important mechanism in the development of diabetic kidney disease [35].

Vallon and co-workers proposed that in early diabetes, the enhanced reabsorption along the proximal tubules, which is in part attributable to the increase in Na^+^-glucose cotransport, has a primary role in the increase in SNGFR: the enhanced proximal tubular transport lowers the tubuloglomerular feedback signal, i.e., the luminal [Cl^−^] sensed by the macula densa cells, resulting in a feedback-induced increase in SNGFR. That “tubule-centric” hypothesis was supported by micropuncture experiments in rats [36,37]. Predictions of the present human kidney model are consistent with this tubule-centric hypothesis: the enhanced proximal tubular transport in diabetes is predicted to lower luminal [Cl^−^] at the macula densa by 24–27% in the moderate diabetes case, and by 32–52% in the severe diabetes case, compared to the non-diabetic kidney, depending on nephron type. In particular, luminal [Cl^-^] at the macula densa of the superficial nephron is predicted to be 28.3, 21.3, and 18.6 mM in the non-diabetic, moderate diabetes, and severe diabetes cases, respectively. If GFR were set to non-diabetic levels in the moderate and severe diabetes cases, the predicted macula densa luminal [Cl^−^] would be lower at 17.7 and 15.6 mM, respectively. Thus, if tubuloglomerular feedback is present in a human kidney, the resulting vasodilative signal predicted in diabetes could contribute to glomerular hyperfiltration. Even though SNGFR was assumed known *a priori* in the simulations, a negative feedback function can be defined that yields the assumed SNGFR given the predicted luminal [Cl^−^] at the macula densa.

Moreover, the model predicts that, by limiting Na^+^-glucose transport, SGLT2 inhibition significantly increases macula densa luminal [Cl^−^], which may then attenuate glomerular hyperfiltration via tubuloglomerular feedback. In particular, following the administration of a SGLT2 inhibitor to the moderate diabetes model, macula densa luminal [Cl^−^] along the superficial nephron is predicted to be 39.2 mM with the assumed GFR-lowering effect, and 51.4 mM without. The corresponding predictions for the severe diabetes model are 36.4 and 48.1 mM, respectively, with and without the GFR-lowering effect. Similar trends are observed in the juxtamedullary nephrons. These results are consistent with clinical data showing that chronic SGLT2 inhibition induced a reduction in eGFR in type 2 diabetes mellitus patients, even among those with chronic kidney disease [38]. Lowering glomerular hyperfiltration on the single nephron level by SGLT2 inhibition may provide long-term beneficial effects in the diabetic kidney. One question is: To what extent does SGLT2 inhibition reduces tubular Na^+^ transport and oxygen consumption along the nephron? The model predicts that the administration of SGLT2 inhibitors in a diabetic kidney decreases overall T_Na_ by 21.8%, but with most of that reduction occurring via the paracellular pathway and with active T_Na_ decreased by only 3%. 

In sum, we have developed the first computational model of detailed epithelial transport in the kidney of a patient with diabetes. The model predicts that, similar to rodents, diabetes-induced tubular hypertrophy in humans may contribute to glomerular hyperfiltration via a (hypothesized) tubuloglomerular feedback signal [36], which in turn results in major increases in transport load and metabolic demand. The model can be used to assess the effects of other commonly-prescribed medications, such as blockers of the angiotensin II system, on kidney function in diabetes, and to assess the extent to which those effects translate to a kidney with reduced nephron number and impaired function. 

It is noteworthy that the present model has adopted many of the assumptions made in a kidney model of a male rat [14,16,39]. Major sexual dimorphism has been revealed in the abundance of electrolyte transporters, channels, and claudins in male and female rodents [40,41]. Findings by Veiras et al. indicated that, compared with male rat nephrons, female rat nephrons exhibit lower activities of major Na^+^ and water transporters along the proximal portion of the renal tubule (proximal tubule), resulting in significantly larger fractional delivery of Na^+^ and water to the downstream nephron segments in female kidneys. Along the distal nephron segments, the female kidney exhibits a higher abundance of key Na^+^ transporters, relative to male, resulting in similar urine excretion between the sexes. Some of these sex differences in rodent kidney function likely translate to humans. Men are known to have higher blood pressure compared to age-matched women before menopause [42,43]. Given the kidney’s key role in blood pressure control, the observed sex differences in hypertension prevalence may be attributable, in part, to differences in kidney structure and function [44,45,46]. Major differences between the rodent and human kidneys notwithstanding, a computational model of the kidney of a woman, inspired by renal transport pattern in the female rodents [47,48,49], may be useful in analyzing kidney function of a female patient with diabetes and in identifying the mechanisms that explain the observed sex differences in diabetic kidney disease [50].

## 4. Materials and Methods

**Figure 5:**
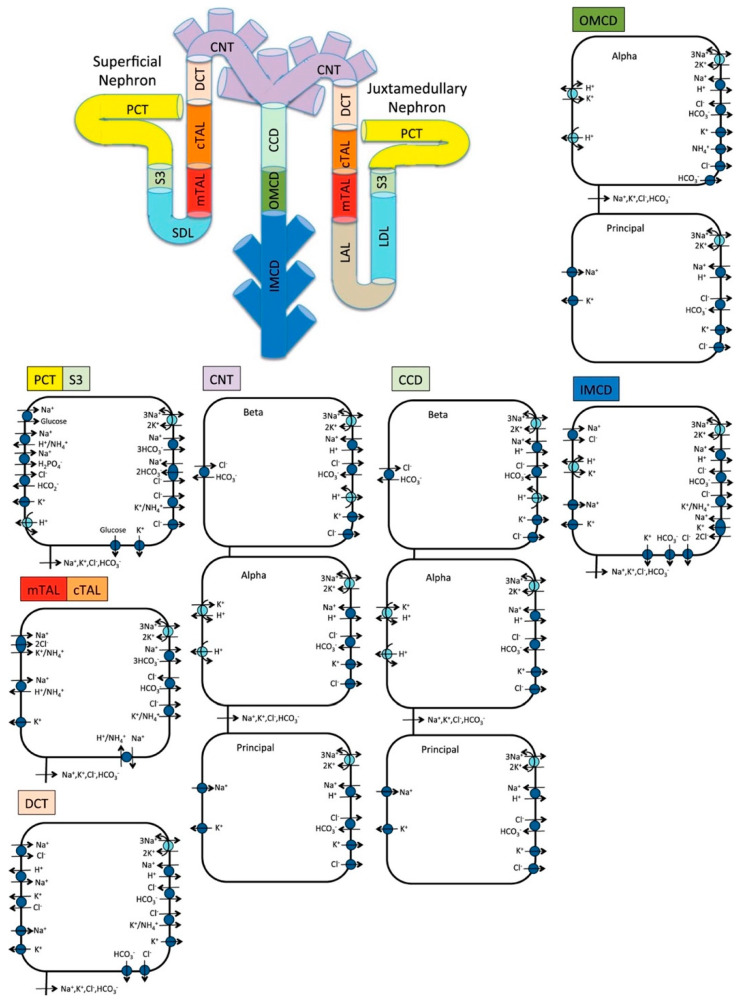
Schematic diagram of the nephron system (not to scale). The model includes 1 representative superficial nephron and 5 representative juxtamedullary nephrons, each scaled by the appropriate population ratio. Only the superficial nephron and one juxtamedullary nephron are shown. Along each nephron, the model accounts for the transport of water and 15 solutes (see text). The diagram displays only the main Na^+^, K^+^, and Cl^−^ transporters. mTAL, medullary thick ascending limb; cTAL, cortical thick ascending limb; DCT, distal convoluted tubule; PCT, proximal convoluted tubule; CNT, connecting duct; CCD, cortical collecting duct; SDL, short or outer-medullary descending limb; LDL/LAL, thin descending/ascending limb; OMCD, outer-medullary collecting duct; IMCD, inner-medullary collecting duct.

We previously developed an epithelial cell-based model of solute transport along the nephrons of a human kidney [10,25]. In this study, we extend that model to simulate a diabetic kidney. The model represents six classes of nephrons: a superficial nephron, which turns at the outer-inner medullary boundary, and five juxtamedullary nephrons, which reach into differing levels of the inner medulla. The superficial nephrons account for 85% of the nephron population, and extend from the Bowman’s capsule to the papillary tip. The remaining 15% of the nephrons are juxtamedullary nephrons that possess loops of Henle that reach to different depths in the inner medulla; most of the long loops turn within the upper inner medulla. Each model nephron is represented as a tubule lined by a layer of epithelial cells, with apical and basolateral transporters that vary according to cell type. The model assumes that the connecting tubules coalesce successively within the cortex, resulting in a ratio of loop-to-cortical collecting duct of 10:1 [51]. The model collecting ducts traverse through the outer medulla, and when they reach the inner medulla, the collecting ducts again coalesce successively. A schematic diagram for the model is shown in Figure 5. In a non-diabetic kidney, single-nephron glomerular filtration rate (SNGFR) is set to 100 and 133 nL/min for the superficial and juxtamedullary nephrons, respectively. Assuming a total of 1 million nephrons in each kidney, this yields a single-kidney GFR of 105 mL/min. 

The model accounts for the following 15 solutes: Na^+^, K^+^, Cl^−^, HCO_3_^−^, H_2_CO_3_, CO_2_, NH_3_, NH_4_^+^, HPO_4_^2−^, H_2_PO_4_^−^, H^+^, HCO_2_^−^, H_2_CO_2_, urea, and glucose. The model is formulated for steady state and consists of a large system of coupled ordinary differential equations and algebraic equations [10]. Model solution describes luminal fluid flow, hydrostatic pressure, luminal fluid solute concentrations and, with the exception of the descending limb segment, cytosolic solute concentrations, membrane potential, and transcellular and paracellular fluxes. Model parameters that describe a non-diabetic kidney can be found in Ref. [10].

### 4.1. Glucose Transport in the Proximal Tubule

Under physiological conditions, the filtered load of glucose is fully reabsorbed in the proximal tubule via SGLTs on the apical side and glucose transport facilitators (GLUT) on the basolateral side. The proximal convoluted tubule expresses the high-capacity, low-affinity transporter SGLT2 together with basolateral GLUT2, whereas the S3 segment expresses the lower capacity, higher affinity transporter SGLT1 and basolateral GLUT1. SGLT1 expression level was chosen such that following SGLT2 inhibition, the non-diabetic model predicts SGLT1-mediated transport of ~60% of the filtered glucose [52]. The modeling of glucose and Na^+^ fluxes across SGLT2 and SGLT1 cotransporters, and glucose fluxes across GLUT1 and GLUT2 have been described in our previous studies [14,16,17,39].

### 4.2. Simulating a Diabetic Kidney

We simulate two diabetic conditions, one in which plasma glucose is elevated from the non-diabetic value of 5 mM to 8.6 mM (which we refer to as the “moderate diabetes case”) and the other in which plasma glucose is 20 mM (the “severe diabetes case”). Diabetes induces renal hypertrophy, hyperfiltration, and alterations in transporter expression. Thus, in addition to increasing plasma glucose concentration, we simulated diabetic conditions by increasing SNGFR by 27 and 10% [4] in superficial and juxtamedullary nephrons, respectively, in both the moderate and severe cases. To simulate tubular hypertrophy, we increased tubular diameter and length of the proximal tubules by 10% in the moderate case and by 28% in the severe case; and we increased the diameter and length of the distal segments by 18 and 7%, respectively, in the moderate case, and by 42 and 7%, respectively, in the severe case [17,53]. Furthermore, the activity of SGLT2 is upregulated by +38%, GLUT2 by +50%, and NKCC by +10%. SGLT1 activity is downregulated by −33%. Na/K-ATPase activity is increased by +10% along all nephron segments, except the thick ascending limb and inner medullary collecting duct. Along the thick ascending limbs, Na/K-ATPase activity is increased by +20%. For the inner medullary collecting duct, Na/K-ATPase activity is increased by +50% along the initial 2/3 and +150% along the remainder of the segment in the moderate diabetes case, and by +150% along the entire segment in severe diabetes. Transcellular water permeability is enhanced along the cortical and inner-medullary collecting duct segments by 55 and 40%, respectively [17]. 

## Data Availability

Computer code will be made publicly available upon the acceptance of this manuscript.
